# Assigning *Culicoides* larvae to species using DNA barcoding of adult females and phylogenetic associations

**DOI:** 10.1186/s13071-022-05479-1

**Published:** 2022-09-30

**Authors:** Tao Jin, Claudia Husseneder, Lane Foil

**Affiliations:** 1 Department of Entomology, Agricultural Experiment Station, Louisiana State University Agricultural Center, Baton Rouge, LA USA; 2 Agricultural Experiment Station, Louisiana State University Agricultural Center—Bob R. Jones-Idlewild Research Station, Clinton, LA USA

**Keywords:** Tree hole habitats, *Culicoides* larvae, DNA barcoding

## Abstract

**Background:**

*Orbivirus*-induced hemorrhagic diseases cause high mortality in wild and captive white-tailed deer in North America. The role of different *Culicoides* species in *Orbivirus* transmission outside of areas of intensive animal production has not been established. At our study location, bluetongue virus (BTV) RNA-positive female *Culicoides debilipalpis* pools have been detected annually since 2012 when BTV transmission was noted in a captive deer herd. Identifying specific larval habitats of suspected vectors at active transmission sites is crucial both for identifying the source of the vectors and for subsequently planning intervention actions. Since *C. debilipalpis* larvae are known to develop in tree holes, this study was designed to use DNA barcoding to identify larvae collected from tree holes.

**Methods:**

Adult female *Culicoides* were collected using light or emergence traps and morphologically identified to 11 species. *Culicoides sonorensis* were also obtained from a laboratory colony. Substrate was collected from tree holes and flooded with water to harvest floating larvae. Total DNA from three to seven adult females per species and 19 larvae was extracted. Two loci of the nuclear 18S ribosomal RNA (rRNA) gene, one locus each of the mitochondrial cytochrome oxidase subunit I (COI) gene and the nuclear 28S rRNA gene were amplified using loci-specific primers.

**Results:**

All 61 adults were sequenced at each of the four loci under study. Since no single locus delineated all putative species and the COI locus yielded unreliable pseudogenes for two individuals of *C. arboricola*, sequences of all four loci were concatenated to maximize species separation and allow for larval association with identified adults. Sixteen larvae were clearly assigned to species based on DNA barcoding and phylogenetic results. Multiple larvae were assigned to each of the *C. debilipalpis* clade, the *C. villosipennis* clade, the *C. arboricola* clade and the *C. nanus* clade.

**Conclusions:**

Of the approximately 62 species described in the southeast USA, 21 have now been barcoded and sequences are publicly available. In this study, we constructed a database composed of species-specific sequences of adult *Culicoides* and then identified larvae to species by matching their corresponding sequences with adults. Since *Culicoides* larvae are difficult to identify, using DNA barcoding to facilitate larval habitat surveys can be a valuable tool.

**Graphical Abstract:**

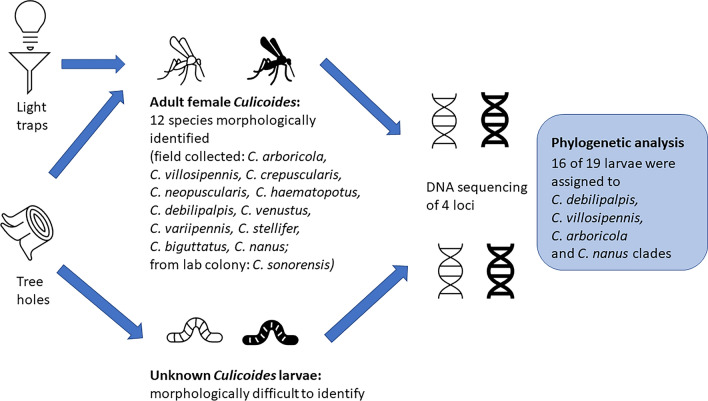

**Supplementary Information:**

The online version contains supplementary material available at 10.1186/s13071-022-05479-1.

## Background

Ceratopogonidae (Insecta: Diptera) is a large family of flies containing approximately 123 genera and over 6200 described species with a worldwide distribution [1]. Flies of the genus *Culicoides* are of medical and veterinary interest because they are biological vectors of > 50 arboviruses [2]. More than 1,350 species have been described worldwide within the genus *Culicoides* [3], including 33 subgenera and 38 unplaced groups consisting of informal subgenera with representative species [4, 5].

Among the arboviruses that have been isolated from *Culicoides* are the Oropouche virus, bovine ephemeral fever virus, bluetongue virus (BTV) and epizootic hemorrhagic disease virus (EHDV). The orbiviruses EHDV and BTV cause a hemorrhagic disease in small ruminants, including some species of deer. The mortality rates are extremely high (90%) in infected white-tailed deer [6], but cattle normally do not show clinical signs for BTV infection and are considered to be reservoirs of BTV. Tabachnick [7] summarized the list of *Culicoides* species considered to be primary vectors of BTV in different areas of the world. Females of *Culicoides sonorensis* (Wirth and Jones, 1957) are thought to be the primary vectors of BTV in certain areas of intensive livestock production in the USA, but the epizootiology of BTV in many other areas of the USA is poorly described.

There have been numerous field studies showing the transmission of BTV and EHDV where specimens of *C. sonorensis* were not collected. In Alabama, BTV RNA products were detected in female *Culicoides stellifer* (Coquillett, 1901), which are very common in that area [8]. In southern Florida, *Culicoides insignis* (Lutz, 1913) is considered to be the primary vector of BTV [9, 10]. Smith et al. [11] collected specimens of *Culicoides debilipalpis* (Lutz, 1913)*, Culicoides stellifer, Culicoides biguttatus* (Coquillett, 1901)*, Culicoides venustus* (Hoffman, 1925) and *Culicoides arboricola* (Root and Hoffman, 1937) from white-tailed deer in Georgia at an enzootic site for hemorrhagic disease, but did not capture a single specimen of *C. sonorensis*. Similarly, Becker et al. [12] collected specimens of *Culicoides* in Louisiana where BTV transmission was shown in white-tailed deer and cattle using competitive enzyme-linked immunosorbent assays and virus neutralization tests. Specimens from *Culicoides crepuscularis* (Malloch, 1915)*, C. debilipalpis, Culicoides haematopotus* (Malloch, 1915) and *Culicoides furens* (Poey, 1853) were found to be PCR-positive for BTV; no specimens of *C. sonorensis* were captured throughout the study. More recently, researchers in Florida have implicated members of *C. venustus* and *C. stellifer* as vectors for EHDV after detecting positive pools from field-collected specimens during an epizootic of EHDV at white-tailed deer farms in Florida where individuals of *C. sonorensis* were not detected [13].

In Louisiana, Becker et al. [14] conducted a prospective study on transmission of orbiviruses in captive deer and cattle at the Bob R. Jones Idlewild Research Station near Clinton, Louisiana, during an epizootic in 2012. Of 14 species captured, BTV viral nucleic acid was detected in pools of three different species of midges: *C. crepuscularis*, *C. debilipalpis* and *C. stellifer.* Among these three species, *C. debilipalpis* has been incriminated as a vector of BTV and EHDV in Louisiana and other locations in the southeastern USA [11, 12, 14–16].

If integrated pest management programs for controlling the vectors of BTV and EHDV are to be developed, identification of the larval habitats of potential vectors would be important information for direct targeting attempts. McDermont and Lysyk [17] described three general larval habitats of *Culicoides*: semiaquatic mud (puddle and wastewater ponds), soil (swamp) and tree holes; they also described methods for collecting immatures. The larval habitat of *C. debilipalpis* has been shown to be tree holes on multiple occasions [18].

Another reason to identify larval habitats and the different species that develop in those niches would be to have an alternate inventory of potential vector species in addition to adult trapping methods as different trap methods do not always account for the species that are present. For example, Carpenter et al. [19] found that populations of *Culicoides chiopterus* (Meigen, 1830) were substantially underestimated using light traps versus drop traps with live sheep, and that this trap bias had resulted in the underestimate of the importance of *C. chiopterus* as a vector of BTV. Subsequently, Viennet et al. [20] captured 17 species of *Culicoides* in a study comparing animal-baited traps to light traps, and reported the capture of 10 species with drop traps and 15 species with light traps, with abundance higher in the drop traps. Therefore, it is important to know if there are species that are not represented when adult trapping methods are used for potential vectors.

Although the larvae of many of the species of *Culicoides* in North America have been described [18, 21], identification is both time consuming and technical even if the described instar is isolated. One method for identifying the habitats of immature *Culicoides* is by using emergence traps in situ or by bringing habitat samples into a laboratory and placing them in emergence traps. One limitation to this approach would be that the larvae of different species may not survive and emerge as adults under those conditions. A more direct approach would be to identify larvae isolated from immature habitats. Since larvae are difficult to identify when using morphological characters, using molecular approaches and matching the larvae to sequences of adults is a valid approach. These methods have been validated in studies in Japan and Senegal [22, 23], but similar studies have not been conducted in Louisiana.

The first objective of this study was to establish a combination of sequences from different loci (mitochondrial cytochrome oxidase subunit I [COI], 18S ribosomal RNA [rRNA] and 28S rRNA genes) as DNA barcodes that can discriminate between adults of *Culicoides* species from a Louisiana study location and then conduct phylogenetic analyses. The second objective was to use these barcodes to identify larvae collected from tree holes in the same area to the species level.

## Methods

### Collection of culicoides

Substrate samples (approximately 100-500 g) were collected from tree holes at the Bob R. Jones-Idlewild Research Station (30.817954 N,−90.97324 W) near Clinton, Louisiana. Half of substrate sample was used to directly collect *Culicoides* larvae as follows. Approximately 20 g of substrate at a time was placed into a small metal pan (250 × 450 mm) and 25 ml of distilled water was added to the pan; live *Culicoides* larvae were collected and placed into 1.5-ml Eppendorf tubes containing 95% ethanol and then stored at −20 °C.

The other half of the sample was placed into mosquito breeder containers (Bioquip Products Inc., Compton, CA, USA; catalog #1425). The containers were kept in an insectary or incubation room where the humidity (75%) and temperature (27 °C) was kept constant. Adult insects, including emerged *Culicoides*, were collected weekly by placing the containers in a freezer at − 20 °C for 3 min to knock down the flies.

In an attempt to establish a complete synoptic collection of the species of *Culicoides* that were native to the study location, additional adults were collected at the Bob R. Jones-Idlewild Research Station using miniature CDC black light traps with UV 4 Watt tube (model 512; John W. Hock Co., Gainesville, FL, USA) baited with 2 kg of dry ice in igloo containers that were deployed before dusk and collected the next morning after sunrise weekly from May through November 2017. The traps were hung approximately 1.5 m above the ground at three different locations. Members of the genus *Culicoides* that emerged from the tree hole samples and those collected by light traps (summarized in Table 1) were sorted by species through examination of wing patterns using the keys of Blanton and Wirth [18]; adult *Culicoides* were placed into 1.5-ml Eppendorf vials containing 95% ethanol. Adult specimens of *C. sonorensis* were obtained from the colony maintained at the Arthropod-borne Animal Diseases Research laboratory in Manhattan, Kansas to use in validating methods for phylogenetic comparisons. In addition to the emerging adults, 24 larvae were collected from tree holes and stored in 95% ethanol at − 20 °C.

**Table 1:** The collection sources of the specimens of adult *Culicoides*. All of the larvae used in this study were collected from tree holes

*Culicoides* species	Collection source
*C. arboricola*	Emerged from tree hole
*C. villosipennis*	Emerged from tree hole
*C. crepuscularis*	Light trap
*C. neopulicaris*	Light trap
*C. haematopotus*	Light trap
*C. debilipalpis*	Emerged from tree hole
*C. venustus*	Light trap
*C. sonorensis*	Insect colony
*C. variipennis*	Light trap
*C. stellifer*	Emerged from tree hole
*C. biguttatus*	Light trap
*C. nanus*	Emerged from tree hole

### DNA extraction

Total DNA from three to seven adult females per species and from the 19 larvae was extracted using the DNeasy Blood & Tissue Kit (Qiagen, Hilden, Germany) according to the manufacturer’s protocol. Each single adult or larval specimen was thoroughly homogenized in 180 µl ATL buffer (Qiagen) in 1.5-ml microcentrifuge tubes (Eppendorf, Hamburg, Germany) with a sterile pestle (VWR International, Radnor, PA, USA) attached to a cordless motor (VWR International). Total DNA was eluted in a 50-μl volume in AE Buffer (Qiagen). The elutions were quantified on a Qubit 4 Fluorometer (Thermo Fisher Scientific, Waltham, MA, USA) with the Qubit dsDNA BR Assay Kit (Invitrogen, Thermo Fisher Scientific,) resulting in a concentration range of 0.25 ng/µl to approximately 1.5 ng/µl.

### DNA barcoding by PCR and Sanger sequencing

Two loci of the nuclear 18S rRNA gene and one locus each of the COI gene and the nuclear 28S rRNA gene were amplified in a 25-µl volume PCR cocktail containing 2 µl genomic DNA template and the primers at a final concentration of 0.2 µM. All of the PCR reactions were conducted using OneTaq Hot Start 2X Master Mix (New England Biolabs, Ipswich, MA, USA) and with cycling conditions that included an initial denaturation at 94 °C for 2 min and a final extension at 68 °C for 5 min. Detailed descriptions of the specific cycling conditions for each series of PCRs are shown in Table 2. All primers were fused with M13 tails at the 5′ end for subsequent Sanger sequencing. Amplicons were run in 1% agarose gels together with the E-Gel™ Low Range Quantitative DNA Ladder (Invitrogen, Thermo Fisher Scientific) to confirm product sizes, then purified by the QIAquick PCR purification kit (Qiagen) and finally eluted in 40 µl Low-EDTA TE Buffer (pH 8.0; Quality Biological Inc., Gaithersburg, MD, USA). The concentrations of PCR products were measured on the Qubit 4 Fluorometer (Thermo Fisher Scientific) with Qubit dsDNA HS Assay Kit (Invitrogen, Thermo Fisher Scientific). Amplicons (5–12 ng) were sent to the LSU Genomics Facility for bidirectional Sanger sequencing on an ABI 3130xl Genetic Analyzer (Applied Biosystems, Thermo Fisher Scientific).

**Table 2:** Loci, primer sequences and PCR conditions

Loci	Primer sequences	PCR conditions
18S rRNA gene (region 1)	M13F-18S ai (5′-*TGTAAAACGACGGCCAGT* CCTGAGAAACGGCTACCACATC-3′) M13R-18S bi (5′-*CAGGAAACAGCTATGAC* GAGTCTCGTTCGTTATCGGA-3′) [55]	Denaturation at 94 °C for 30 s, annealing at 52 °C for 45 s and extension at 68 °C for 1 min 15 s, for 34 cycles
18S rRNA gene (region 2)	M13F-NF1 (5′-*TGTAAAACGACGGCCAGT* GGTGGTGCATGGCCGTTCTTAGTT-3′) M13R-18Sr2b (5′-C*AGGAAACAGCTATGAC* TACAAAGGGCAGGGACGTAAT-3′) [56]	Denaturation at 94 °C for 30 s, annealing at 54 °C for 45 s and extension at 68 °C for 30 s, for 34 cycles
COI	M13F-CIJ1718 (5′-*TGTAAAACGACGGCCAGT* GGAGGATTTGGAAATTGATTAGT-3′) M13R-CIN2191 (5′-*CAGGAAACAGCTATGAC* CAGGTAAAATTAAAATATAAACTTCTGG-3′) [43]	Denaturation at 94 °C for 30 s, annealing at 46 °C for 45 s and extension at 68 °C for 40 s, for 34 cycles
28S rRNA gene (D1D2)	M13F-C′1 (5′-*TGTAAAACGACGGCCAGT* ACCCGCTGAATTTAAGCAT-3′) M13R-D′2 (5′-*CAGGAAACAGCTATGAC* TCCGTGTTTCAAGACGGG-3′) [34]	Denaturation at 94 °C for 30 s, annealing at 50 °C for 45 s and extension at 68 °C for 50 s, for 34 cycles

*COI* Mitochondrial cytochrome oxidase subunit I

### Sequence analysis

Sequences were analyzed with Geneious Prime 2019.0.3 software. The sequences were first trimmed with an error probability limit ≤ 0.03, and then forward and reverse sequences were assembled into single contigs. All contigs of each gene region were aligned respectively and then truncated to the same length with gap placement, resulting in sequence lengths of 877 bp and 378 bp for the two loci of the 18S rRNA gene (18S region 1 and region 2, respectively), 713 bp for the fragment of 28S rRNA gene and 426 bp for the fragment of COI gene. The four loci were then manually concatenated. MUSCLE (v.3.8.425) was used for alignment with the following settings: kmer4_6 (iteration 1) and pctid_kimura (subsequent) for distance measure; UPGMB for clustering method; pseudo for tree rooting; CLUSTALW for sequence weighting scheme [24]. The alignment files were exported in PHYLIP format. PartitionFinder2 [25] was used to find the best-fit partitioning schemes for the concatenated contigs with the following options: branch lengths = linked; models = all; model_selection = AICc; search = greedy [26, 27]. The output partition file was exported in NEXUS format. Maximum likelihood (ML) trees were constructed using IQ-TREE web server [28] with the auto substitution model [29] and ultrafast bootstrap analysis [30] with 10,000 bootstrap alignments. The bootstrap value of 85 was set as the omission threshold. For generating the phylogenetic tree of concatenated contigs, we also uploaded the partition file from PartitionFinder2 to IQ-TREE server [31]. One to six sequences corresponding to the individual locus of each species with the highest percentage of identities with our sequences were selected from the NCBI database as the reference sequences via Basic Local Alignment Search Tool (BLAST) search. It should be noted that NCBI database does not contain references for all four loci for some species. *Dasyhelea* sp. was used as the outgroup in the phylogenetic trees constructed from region 1 of 18S rRNA and COI gene fragments as well as concatenated sequences; *Dixella cornuta* and *Forcipomyia pluvialis* represented the outgroups in the phylogenetic trees constructed from region 2 of the 18S rRNA and 28S rRNA genes, respectively. The phylogenetic tree of concatenated sequences was demonstrated and manipulated using Interactive Tree Of Life (iTOL) [32], and the phylogenetic trees of each individual locus were demonstrated and manipulated using Figtree [33].

## Results

### Species identification of adults

The 61 adult specimens that were identified to species based on morphology represented 12 species across nine subgenera and one unplaced group. All 61 taxa were sequenced at the four studied loci (Table 3) [4]. Regions 1 and 2 of the 18S rRNA gene displayed 83 variant sites (9.34% of all sites) with 38 parsimony informative sites (sites with at least two-character states that occur at least in two sequences), and 85 variant sites (22.02% of all sites) with 46 parsimony informative sites, respectively. The 28S rRNA and COI genes had higher percentages of variant sites, showing 132 variant sites (18.36% of all sites) with 80 parsimony informative sites, and 187 variant sites (43.90% of all sites) with 180 parsimony informative sites, respectively. The mitochondrial COI gene had a higher proportion of parsimony-informative sites than the nuclear ribosomal genes. The fragment of the COI gene only contained single nucleotide polymorphisms (SNPs), while the other three loci contained both SNPs and insertion-deletion mutations.

**Table 3:** *Culicoides* subgenera and species represented in this study, identified by adult wing morphology and DNA sequence data for four loci

Subgenus	*Culicoides* species	GenBank accession number of samples collected in this study			
		18S (region 1)	18S (region 2)	COI	28S
*Amossovia* Glukhova	*C. arboricola *(*n* = 5)	MT335887-335891	MT335954-335958	MT328916-328920	MT336023-336027
	*C. villosipennis *(*n* = 3)	MT335951-335953	MT336018-336020	MT328980-328982	MT336087-336089
*Beltranmyia* Vargas	*C. crepuscularis *(*n* = 7)	MT335899-335905	MT335966-335972	MT328928-328934	MT336035-336041
*Culicoides* Latreille	*C. neopulicaris *(*n* = 4)	MT335920-335923	MT335987-335990	MT328949-328952	MT336056-336059
*Diphaomyia* vargas	*C. haematopotus *(*n* = 4)	MT335911-335914	MT335978-335981	MT328940-328943	MT336047-336050
*Haematomyidium* Goeldi	*C. debilipalpis* (*n* = 5)	MT335906-335910	MT335973-335977	MT328935-328939	MT336042-336046
*Hoffmania* Fox	*C. venustus* (*n* = 5)	MT335946-335950	MT336013-336017	MT328975-328979	MT336082-336086
*Monoculicoides* Khalaf	*C. sonorensis* (*n* = 5)	MT335924-335928	MT335991-335995	MT328953-328957	MT336060-336064
	*C. variipennis *(*n* = 5)	MT335941-335945	MT336008-336012	MT328970-328974	MT336077-336081
*Oecacta* Poey	*C. stellifer *(*n* = 5)	MT335935-335940	MT336002-336007	MT328964-328969	MT336071-336076
*Silvaticulicoides* Glukhova	*C. biguttatus *(*n* = 7)	MT335892-335898	MT335959-335965	MT328921-328927	MT336028-336034
Unplaced	*C. nanus *(*n* = 5)	MT335915-335919	MT335982-335986	MT328944-328948	MT336051-336055

### Individual gene trees

Separate evaluation of each gene region showed a different resolution power of loci for species differentiation. While the intraspecific variation in sequence similarities was comparatively high for all loci (98.84–99.98%), the mean sequence similarity among species was highest for the 18S-1 gene (98.68%), followed by the 18S-2 (95.63%) and 28S genes (95.55%) (Additional file 1: Table S1). The COI gene showed the highest species resolution, i.e., the greatest difference between intra- and interspecific sequence similarity (98.84% vs 81.55%). The differences in resolution power of the loci are reflected in the topology of the ML trees of *Culicoides* spp. (Additional file 2: Figure S1, Additional file 3: Figure S2, Additional file 4: Figure S3, Additional file 5: Figure S4). Due to mostly identical sequences, *C. arboricola* and *C. villosipennis* (Root and Hoffman, 1937), and *C. sonorensis* and *Culicoides variipennis* (Coquillett, 1901) could not be differentiated solely by their 18S rRNA gene sequences.

The 28S rRNA gene contained slightly more interspecific variant sites than the two loci of the 18S rRNA gene and was therefore a more reliable locus to effectively differentiate closely-related species such as *C. arboricola* and *C. villosipennis*. However, no single locus could effectively discriminate between the recently taxonomically separated sibling species *C. sonorensis* and *C. variipennis*, particularly when intraspecific variation was increased by incorporating reference sequences from the NCBI GenBank from different geographical regions. All other species were more effectively delineated by the COI gene than by the ribosomal genes since the former contained the most interspecific variations. Unfortunately, in two of the five individuals morphologically identified as *C. arboricola*, the forward and reverse sequences did not match (only 85% identity). Moreover, while the NCBI database top match of the one-directional sequences from those two specimens was *Culicoides* sp. (99.3%), the other directional sequences matched to *C. arboricola* with 98.2% similarity.

### Concatenated species tree

**Fig. 1:**
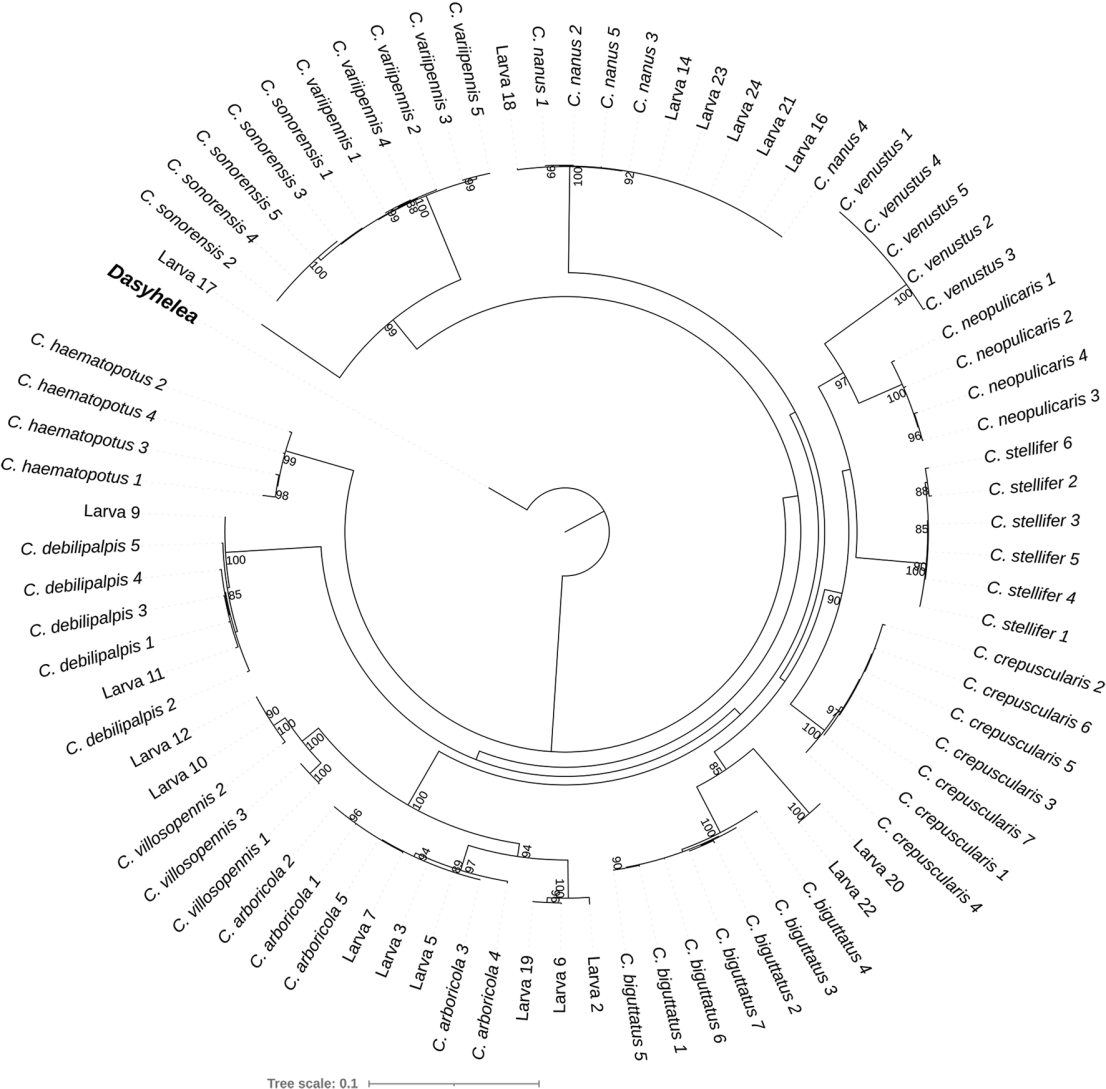
The maximum likelihood (ML) phylogenetic tree of *Culicoides* adults identified to species and of larvae resulting from the concatenated sequences of the four studied loci, with the ceratopogonid *Dasyhelea* sp. (indicated in bold) as the outgroup. Confidence values at branches are based on ultrafast bootstrap analysis with 10,000 bootstrap alignments

Since no single locus delineated all putative species and the COI locus yielded unreliable pseudogenes for two individuals of *C. arboricola*, the sequences of all four loci were concatenated to maximize species separation and allow for larval association with identified adults. The ML tree resulting from the concatenated sequences (Fig. 1) with the ceratopogonid *Dasyhelea* sp. as the outgroup recovered monophyletic groupings of all species based on adult morphology and, moreover, a topology consistent with the current taxonomy. Species from the same morphological subgenus grouped together, such as *C. sonorensis* and *C. variipennis*, as well as *C. arboricola* and *C. villosipennis*. *Culicoides venustus* and *Culicoides neopulicaris* (Wirth, 1955) were recovered in a single clade, which is consistent with previous results [34, 35]. The subgenus *Monoculicoides* was shown to be the most distant taxon among the whole ingroup, as previously described [34, 35].

Of 24 larvae, 19 were successfully mounted and sequenced; of the latter, 16 larvae could be clearly assigned to species based on DNA barcoding and phylogenetic results. Larva #9 and #11 were recovered within the *C. debilipalpis* clade; larva #10 and #12 were recovered within the *C. villosipennis* clade. Three larvae were recovered within the *C. arboricola* clade (larva #3, #5 and #7), and larva #2, #6 and #19 were recovered as monophyletic in a clade sister to the *C. arbicola* clade. Larva #14, #16, #21, #23 and #24 were recovered within the *C. nanus* (Root and Hoffman, 1937) clade, and larva #18 was recovered as a sister taxon. The short branch lengths indicate that larva #18 is likely also a member of *C. nanus*. In contrast, larva #20 and #22 were recovered as monophyletic and sister to the *C. biguttatus* clade. Larva #17 was recovered as sister to the *C. variipennis* + C*. sonorensis* clade but was separated from the *C. sonorensis*/*C. variipennis* clade with high node support.

## Discussion

In this study, we used four loci from three genes to verify the morphologically identified *Culicoides* specimens belonging to 12 species from nine subgenera and one unplaced group. All of the species formed distinct clusters in the phylogenetic trees that were in agreement with morphological species determination. The 18S rRNA gene sequences provided sufficient resolution to differentiate the species at the subgenus level but could not discriminate the sibling species from the same subgenus or species complex. Similarly, in a previous study, 18S rRNA gene markers alone could not discriminate between *Culicoides obsoletus* (Meigen, 1818) and *Culicoides scoticus* (Downes and Kettle, 1952) or identify sibling species of *Culicoides pulicaris* (Linnaeus, 1758) [36]. Even though, both the 28S rRNA and COI genes could be used to differentiate all the species, the COI gene had greater resolution power than the 28S rRNA gene, as previously reported for the *C*. *obsoletus/scoticus* complex [37]. For example, the use of COI sequences detected subtle intraspecific differences in some species, such as *C. stellifer.* However, the larger-than-expected sequence differences observed within *C. arboricola* may be attributed to the existence of nuclear mitochondrial pseudogenes [38]. Overall, the relationships among the clades in the phylogenetic tree based on COI sequences were consistent with those reported in a previous study [39] placing *C. neopulicaris*, *C. stellifer* and the *C. sonorensis*/*variipennis* complex in one group and *C. haematopotus*, *C. debilipalpis* and *C. arboricola* into a separate group. Although COI markers proved useful for species delineation due to their resolution power, the co-amplification of rare pseudogenes with the orthologous COI gene in at least one species (*C. arboricola*) may falsely mimic intraspecific differentiation [38]. Therefore, we combined the information of four loci to mitigate the disadvantage of any single locus.

DNA barcoding has been widely used to characterize *Culicoides* species, their phylogeny and distribution worldwide. For example, the COI gene was used to confirm previously described species and discover cryptic species belonging to subgenus *Culicoides* Latreille in Denmark [40] and Spain [41], as well as the *C*. *pulicaris* complex in Turkey [42]. The phylogenetic status of *Culicoides imicola* (Kieffer, 1913), which is a vector of BTV, was studied with the COI gene marker in Portugal, Rhodes and Israel [43] and South Africa [44]. Another vector, *C. obsoletus*, was characterized within the *C. obsoletus*/*scoticus* complex using COI markers for flies collected in Europe [37]. Augot et al. [34] used concatenated markers (COI and 28S rRNA genes) to conduct phylogenetic analysis of 68 specimens belonging to 42 *Culicoides* species from three continents collected from 2009 to 2010. Barcoding was the only effective method to differentiate sibling species, such as *C. obsoletus* and *C. scoticus,* when morphological identification of specimens was difficult [45]. Molecular methods, in combination with morphological determination, have been used to verify identity of described species in Sweden [46] and Tunisia [47], to discover new species [48, 49], study the species composition [50] and screen the potential BTV vectors in the *obsoletus* complex in France [51].

Tabachnick [7] summarized the list of *Culicoides* species considered to be primary vectors of BTV in different areas of the world; *C. variipennis* and *C. insignis* were accepted as the primary vectors of BTV in the USA and the Caribbean. At that time, *C. variipennis* was thought to have populations refractory to BTV infection in certain areas of North America. Subsequently, Tabachnick [52] and Holbrook et al. [53] conducted morphological and genetic studies which identified *C. variipennis* as a complex of three separate species, namely the competent vector *C. sonorensis* as well as *C. variipennis* and *Culicoides occidentalis* (Wirth and Jones, 1957), with both of the latter shown to be refractory to BTV infection.

Blanton and Wirth’s comprehensive treatment of the “*Culicoides* of Florida” [18] included descriptions of 62 species and their habitats. Of those 62 species, 21 have now been barcoded and sequences are publicly available. The COI gene was used along with other three loci to identify species of *Culicoides* within the *C. variipennis* complex from 25 states across the USA [54]. Martin et al. [39] barcoded 12 species from central Texas, including *C. neopulicaris*, *C. crepuscularis*, *C. arboricola*, *C. nanus*, *C. debilipalpis*, *C. haematopotus*, *Culicoides edeni* (Wirth and Blanton, 1974), *Culicoides hinmani* (Khalaf, 1952), *Culicoides paraensis* (Goeldi, 1905), *C. stellifer*, *C. sonorensis* and *Culicoides multipunctatus* (Malloch, 1915). Subsequently, we barcoded 12 species using four different loci of three genes. For COI sequences, the percentage of identities for the matches between our data and references for the same species in NCBI ranged from 98 to 100%, except for *C. haematopotus* at 95%.

The identification of adult female *Culicoides* in the USA is primarily initiated by recognition of the distinct wing patterns presented in taxonomic works such as Blanton and Wirth [18]. Subsequently, more certain identification of individuals requires clearing of the specimens and mounting to allow examination of the sensory pit patterns of the antennae, the morphology of the spermathecae, among others. In studies aimed at screening specimens of *Culicoides* for pathogens or for abundance, large numbers of specimens of multiple species are sorted by wing patterns once a synoptic collection is established. However, despite considerable quality control, differentiation of specimens within a complex may not be possible without using molecular identification tools. Therefore, studies contributing to the public databases for genetic sequences of additional species of *Culicoides* should be encouraged.

Although there are descriptions of the majority of the species of *Culicoides* larvae in the USA [18, 21], identification of specimens is both time consuming and technical. In this study we were able to match larvae to barcodes of adult females collected in the locality of the larval collections. In our study, we constructed a database composed of species-specific sequences of adult female *Culicoides* and then characterized the larvae by matching their corresponding sequences with adults. We successfully grouped some larvae into *C. nanus*, *C. debilipalpis* and *C. arboricola* clades. In future studies, combining imaging with barcoding could help expand the knowledge of morphological traits of larvae and allow specimens to be identified by image analysis algorithms.

This study as well as similar studies in both Senegal and Japan all produced reliable matches of adults and larvae gene sequences [23]. For future studies aimed at the identification of larvae, matching the identifications to published sequences for adults could be used to confirm identification of larvae. As the number of barcoded species grows, our ability to rapidly identify larvae from different habitats as well as potential geographic differences in the adults and larvae of recognized species will expand.

## Conclusions

In this study we successfully assigned *Culicoides* larvae to known species of adults by using DNA barcoding of adult females and phylogenetic associations. Since *Culicoides* larvae are difficult to identify, using DNA barcoding to facilitate larval habitat surveys can be a valuable tool. In this study, we were able to verify that tree holes are a potential stable larval habitat of *C. debilipalpis* in a location of active *Orbivirus* transmission. As the number of barcoded species from the southern USA increases, we should be able to identify potential geographic differences in the adults and larvae of different species or undescribed species’ complexes.

## Supplementary Information

**Additional file 1: Table S1.  a**. The average of the intra- specific nucleotide percentage of pairwise identities (mean ± standard deviation [SD], in %) of each adult specimen to all the others within one species at each locus.* n* is the number of the specimens within each species; ‘overall’ is the average value of all the means across all the species for each locus.** b** The average of the inter- specific nucleotide percentage of pairwise identities (mean ± SD, in %) of each species to all the others at each locus. ‘Overall’ is the average value of all the means across all the species for each locus.

**Additional file 2: Table S2. **The average of the inter- specific nucleotide percentage of pairwise identities (mean ± standard deviation%) of each species to all the others at each locus; overall is the average value of all the means across all the species for each locus.

**Additional file 3: Figure S1.** The ML phylogenetic tree based on the sequences of the 18S rRNA gene (region 1) with reference sequences from NCBI (Accession number: LN484108.1 and U48380.1) and *Dasyhelea* sp. as the outgroup. Confidence values at branches are based on ultrafast bootstrap analysis with 10,000 bootstrap alignments.

**Additional file 4: Figure S2.** The ML phylogenetic tree based on the sequences of the 18S rRNA gene (region 2) with reference sequences from NCBI (Accession number: U48380.1 and LN484108.1) and *Dixella cornuta* as the outgroup. Confidence values at branches are based on ultrafast bootstrap analysis with 10,000 bootstrap alignments.

**Additional file 5: Figure S3.** The ML phylogenetic tree based on the 28S rRNA gene sequences with reference sequences from NCBI (Accession number: LN484108.1) and *Forcipomyia pluvialis* as the outgroup. Confidence values at branches are based on ultrafast bootstrap analysis with 10,000 bootstrap alignments.

**Additional file 6: Figure S4.** The ML phylogenetic tree based on the COI gene sequences with reference sequences from NCBI (with Accession numbers) and *Dasyhelea notata* as the outgroup. Confidence values at branches are based on ultrafast bootstrap analysis with 10,000 bootstrap alignments.

## Data Availability

The datasets used and/or analyzed during the current study are available in GenBank of National Center for Biotechnology Information (NCBI) with accession numbers listed in Table 3.

## Abbreviations

BTV: Bluetongue virus
EHDV: Epizootic hemorrhagic disease virus
COI: Mitochondrial cytochrome oxidase subunit I
