# Supplementary material for: Assigning Culicoides larvae to species using DNA barcoding of adult females and phylogenetic associations
Source: Parasit Vectors. 2022 Sep 30;15:349. doi: 10.1186/s13071-022-05479-1 (PMC9526334; doi:10.1186/s13071-022-05479-1)
Supplement: Supplementary file 1 — Additional file 1: Table S1. a. The average of the intra- specific nucleotide percentage of pairwise identities (mean ± standard deviation [SD], in %) of each adult specimen to all the others within one species at each locus. n is the number of the specimens within each species; ‘overall’ is the average value of all the means across all the species for each locus. b The average of the inter- specific nucleotide percentage of pairwise identities (mean ± SD, in %) of each species to all the others at each locus. ‘Overall’ is the average value of all the means across all the species for each locus. [file 13071_2022_5479_MOESM1_ESM.docx]

Additional file 1: Table S1 The average of the intra- specific nucleotide percentage of pairwise identities (mean ± standard deviation%) of each adult specimen to all the others within one species at each locus, n is the number of the specimens within each species; overall is the average value of all the means across all the species for each locus.

| Species | n= | Locus | | | |
| --- | --- | --- | --- | --- | --- |
|  |  | 18S-1 | 18S-2 | 28S | COI |
| *C. arboricola* | 5 | 99.95±0.06 | 100.00 | 99.63±0.37 | 99.88±0.09 |
| *C. biguttatus* | 7 | 100.00 | 99.72±0.22 | 100.00 | 99.20±0.46 |
| *C. crepuscularis* | 7 | 99.97±0.05 | 100.00 | 100.00 | 99.60±0.23 |
| *C. debilipalpis* | 5 | 100.00 | 100.00 | 99.77±0.17 | 99.39±0.26 |
| *C. haematopotus* | 4 | 100.00 | 100.00 | 99.86±0.08 | 97.38±1.40 |
| *C. nanus* | 5 | 100.00 | 100.00 | 100.00 | 99.77±0.15 |
| *C. neopulicaris* | 4 | 99.89±0.11 | 99.82±0.12 | 100.00 | 98.83±0.41 |
| *C. sonorensis* | 5 | 100.00 | 100.00 | 100.00 | 99.53±0.58 |
| *C. stellifer* | 5 | 99.94±0.06 | 100.00 | 99.92±0.07 | 99.16±0.69 |
| *C. variipennis* | 5 | 100.00 | 100.00 | 100.00 | 99.06±0.59 |
| *C. venustus* | 5 | 100.00 | 100.00 | 100.00 | 100.00 |
| *C. villosipennis* | 3 | 100.00 | 100.00 | 100.00 | 94.29±3.87 |
| overall |  | 99.98±0.04 | 99.96±0.09 | 99.93±0.12 | 98.84±1.59 |
