# Supplementary material for: Assigning Culicoides larvae to species using DNA barcoding of adult females and phylogenetic associations
Source: Parasit Vectors. 2022 Sep 30;15:349. doi: 10.1186/s13071-022-05479-1 (PMC9526334; doi:10.1186/s13071-022-05479-1)
Supplement: Supplementary file 2 — Additional file 2: Table S2. The average of the inter- specific nucleotide percentage of pairwise identities (mean ± standard deviation%) of each species to all the others at each locus; overall is the average value of all the means across all the species for each locus. [file 13071_2022_5479_MOESM2_ESM.docx]

Additional file 2: Table S2 The average of the inter- specific nucleotide percentage of pairwise identities (mean ± standard deviation%) of each species to all the others at each locus; overall is the average value of all the means across all the species for each locus.

| Species | Locus | | | |
| --- | --- | --- | --- | --- |
|  | 18S-1 | 18S-2 | 28S | COI |
| *C. arboricola* | 98.84±0.55 | 95.73±1.92 | 96.08±1.57 | 80.47±2.47 |
| *C. biguttatus* | 99.15±0.40 | 95.85±1.02 | 95.86±1.10 | 81.94±2.25 |
| *C. crepuscularis* | 98.94±0.33 | 96.62±1.26 | 95.75±1.20 | 82.55±1.58 |
| *C. debilipalpis* | 98.59±0.31 | 95.65±0.93 | 95.27±0.68 | 80.75±2.16 |
| *C. haematopotus* | 98.18±0.34 | 94.60±0.65 | 95.39±0.72 | 82.14±1.55 |
| *C. nanus* | 98.10±0.42 | 95.88±1.18 | 96.38±1.19 | 78.75±1.30 |
| *C. neopulicaris* | 98.91±0.46 | 96.32±1.45 | 96.01±1.25 | 81.07±2.47 |
| *C. sonorensis* | 98.63±0.57 | 95.01±1.72 | 94.57±1.84 | 83.74±5.40 |
| *C. stellifer* | 98.57±0.31 | 95.83±1.25 | 95.80±0.50 | 82.67±2.44 |
| *C. variipennis* | 98.63±0.57 | 95.01±1.72 | 94.70±1.79 | 83.83±5.38 |
| *C. venustus* | 98.66±0.46 | 95.30±1.48 | 95.05±0.73 | 80.81±1.47 |
| *C. villosipennis* | 98.94±0.53 | 95.73±1.92 | 95.80±1.69 | 79.91±2.97 |
| overall | 98.68±0.52 | 95.63±1.47 | 95.55±1.32 | 81.55±3.16 |
